# Validating Automated Sentiment Analysis of Online Cognitive Behavioral Therapy Patient Texts: An Exploratory Study

**DOI:** 10.3389/fpsyg.2019.01065

**Published:** 2019-05-14

**Authors:** Simon Provoost, Jeroen Ruwaard, Ward van Breda, Heleen Riper, Tibor Bosse

**Affiliations:** ^1^Department of Clinical, Neuro- and Developmental Psychology, Section Clinical Psychology, Amsterdam Public Health Research Institute, Vrije Universiteit Amsterdam, Amsterdam, Netherlands; ^2^Department of Psychiatry, Amsterdam Public Health Research Institute, Amsterdam UMC, Vrije Universiteit Amsterdam, Amsterdam, Netherlands; ^3^GGZ inGeest Specialized Mental Health Care, Amsterdam, Netherlands; ^4^NeedForward Research, Dordrecht, Netherlands; ^5^Centre for Telepsychiatry, Institute of Clinical Research, University of Southern Denmark, Odense, Denmark; ^6^Behavioural Science Institute, Radboud University Nijmegen, Nijmegen, Netherlands

**Keywords:** sentiment analysis and opinion mining, internet interventions, cognitive behavioral therapy (CBT), e-mental health, embodied conversational agent (ECA), automated support, benchmarking and validation, depression

## Abstract

**Introduction:**

Sentiment analysis may be a useful technique to derive a user’s emotional state from free text input, allowing for more empathic automated feedback in online cognitive behavioral therapy (iCBT) interventions for psychological disorders such as depression. As guided iCBT is considered more effective than unguided iCBT, such automated feedback may help close the gap between the two. The accuracy of automated sentiment analysis is domain dependent, and it is unclear how well the technology is applicable to iCBT. This paper presents an empirical study in which automated sentiment analysis by an algorithm for the Dutch language is validated against human judgment.

**Methods:**

A total of 493 iCBT user texts were evaluated on overall sentiment and the presence of five specific emotions by an algorithm, and by 52 psychology students who evaluated 75 randomly selected texts each, providing about eight human evaluations per text. Inter-rater agreement (IRR) between algorithm and humans, and humans among each other, was analyzed by calculating the intra-class correlation under a numerical interpretation of the data, and Cohen’s kappa, and Krippendorff’s alpha under a categorical interpretation.

**Results:**

All analyses indicated moderate agreement between the algorithm and average human judgment with respect to evaluating overall sentiment, and low agreement for the specific emotions. Somewhat surprisingly, the same was the case for the IRR among human judges, which means that the algorithm performed about as well as a randomly selected human judge. Thus, considering average human judgment as a benchmark for the applicability of automated sentiment analysis, the technique can be considered for practical application.

**Discussion/Conclusion:**

The low human-human agreement on the presence of emotions may be due to the nature of the texts, it may simply be difficult for humans to agree on the presence of the selected emotions, or perhaps trained therapists would have reached more consensus. Future research may focus on validating the algorithm against a more solid benchmark, on applying the algorithm in an application in which empathic feedback is provided, for example, by an embodied conversational agent, or on improving the algorithm for the iCBT domain with a bottom-up machine learning approach.

## Introduction

Internet-delivered cognitive behavioral therapy (iCBT) has been found equally effective as face-to-face therapy (Andersson and Cuijpers, 2009). It can be unguided or guided, with guidance being provided by trained volunteers or health professionals, and taking the form of “coaching”, e.g., providing motivation or technical assistance, or “treatment”, e.g., engaging in a therapeutic relationship (Schueller et al., 2017). Thus far, iCBT seems more effective when it includes guidance than when guidance is absent (Richards and Richardson, 2012: Andersson and Titov, 2014). The present study is part of a project in which we explore whether we can bridge the gap between guided and unguided interventions, either completely or partially, through automated support by embodied conversational agents (ECAs), computer-generated characters that can simulate verbal and non-verbal behaviors similar to those used in human face-to-face conversations (Isbister and Doyle, 2004).

When providing support, a number of non-specific factors are considered important. Examples are a good therapeutic alliance, positive expectancy effects of both the patient and the coach, therapeutic competence of the supportive human, and the content of written feedback (Mohr et al., 2011; Schueller et al., 2017; Mol et al., 2018). An important element in many of these factors is “empathy”, i.e., perceiving and understanding others’ affective states and acting accordingly (Paiva et al., 2017), which can contribute to a good therapeutic relationship between a supportive human and patient, both in face-to-face (Keijsers et al., 2000), and online settings (Mohr et al., 2011). Similar to human-human interaction, empathy simulated by an ECA can contribute to the bond between the user and ECA, as research has shown, for example, that empathic ECAs are seen as more trustworthy, likeable, and caring (Brave et al., 2005), and can build and sustain long-term relationships with users (Bickmore and Picard, 2005). For example, a recent study showed how the inclusion of empathy conveyed by a virtual character in a brief intervention for problematic drinking behavior could increase intentions to reduce drinking compared to the intervention without empathy (Ellis et al., 2017). In order to successfully express empathy, ECAs must accurately determine a user’s emotional state, and respond appropriately (Paiva et al., 2017). ECAs have been endowed with a variety of techniques to recognize the emotional state of the users they interact with. Examples are the analysis of facial expressions, body posture, acoustic features of speech, and other types of higher-level non-verbal behavior such as fidgeting, as well as linguistic content. In state-of-the-art ECA approaches, these features are combined to gain an optimal understanding of the user’s emotional state (Hartholt et al., 2013).

Internet interventions, however, are often browser-based with limited possibilities for multi-modal interaction through audio-visual communication. Especially in unguided interventions, interaction is usually limited to patient responses to exercises or questions with multiple-choice (e.g., “Select an activity you will try to do this week”), Likert-scale (e.g., “Rate your mood on a scale of one to ten”), or free text input (e.g., “Please describe how you tried to apply the plan you made last week”). Rather than engaging in dyadic dialogs, as in face-to-face therapy or in the interaction with ECAs that make extensive use of emotion detection (e.g., Swartout et al., 2013; Devault et al., 2014), people who provide guidance in iCBT typically do so by email after a patient has completed one of several intervention modules, and base their messages on the patient’s input in the intervention and guidelines for giving online feedback (Mol et al., 2018). Thus, if we want an ECA to provide guidance similar to humans, it should respond to the scarce human input that is available in a guided internet-based intervention, rather than to information obtained during real-time dyadic interactions, and preferably do so empathically. Input based on a limited set of answers (e.g., rating one’s mood on a scale of one to ten) lends itself well to such a task, but dealing with free text input is more difficult as automated processing of the semantic nature of a text is still far from accurate, and often domain specific. In this paper we focus on the application of a technique that tries to determine the semantic content of a text on a higher level of abstraction, namely sentiment analysis.

Sentiment analysis, often interchangeably used with “opinion mining”, e.g., in the domain of product reviews, aims to identify text that contains sentiment, identify what the sentiment is, and determine the overall polarity (negative or positive) of the text (Pang and Lee, 2008). A sentence such as “I liked the module,” for example, would have a positive valence, while “I did not like the module,” would have a negative one. Broadly speaking, sentiment analysis algorithms are either based on bottom-up machine learning approaches, where algorithms learn to recognize sentiment by looking at example texts that have already been classified, while iteratively adjusting parameter values such that the algorithm’s output matches the predetermined classification, or top-down lexicon-based approaches, where they use pre-specified dictionaries to identify sentiment words (Medhat et al., 2014). Sentiment analysis has been researched extensively in the context of social media, in contexts ranging from predictions in politics (Sobkowicz et al., 2012) to the detection of depression (Wang et al., 2013). It has also been used in the area of ECAs, either by analyzing parsed speech or direct free text input, for example to detect a user’s negative emotional states (see Clavel and Callejas, 2016 for a review). An example of an application in the domain of psychology is the detection of depression in micro-blog posts (Wang et al., 2013).

From a literature review on the use of ECAs in clinical psychology, we learned that even though there have been a number of studies involving ECAs in a supporting role in iCBT contexts, evidence on their effectiveness, and validation of the underlying techniques that are used for the clinical domain remains sparse (Provoost et al., 2017). Clinical psychology is an applied science, and therefore, before novel technologies can be applied in practice, they require a thorough validation and understanding. However, we still know little about the application of sentiment analysis in the context of providing automated guidance in iCBT for depression. Moreover, sentiment analysis can be highly domain specific, considering, for example, the different vocabulary and grammatical engines required to classify newspaper articles versus Twitter messages. Therefore, in this paper we investigate how an existing sentiment analysis algorithm, using a top-down lexicon-based approach, evaluates free text input provided by patients using an iCBT intervention for depression compared to human judges. We further describe the implications of our findings for the applicability of sentiment analysis in clinical practice, and how they can inform further research.

## Materials and Methods

### Design

We conducted an exploratory study in which texts, written by patients during online therapy, were evaluated on overall sentiment and emotional expressiveness. Evaluation was conducted (1) automatically by an algorithm, and (2) manually by a group of human judges. From a set of 493 patient texts, subsets of 75 texts were randomly assigned to each human judge, such that every text received a similar amount of evaluations. Approval for the study was obtained from the Research Ethics Committee of the Faculty of Movement and Behavioral Sciences of the Vrije Universiteit Amsterdam (Reference Number: VCWE-2017-165).

### Participants

A total of *N* = 52 first-year psychology students at Vrije Universiteit Amsterdam were recruited. As part of their curriculum they have to partake in experimental studies for at least 10 h, and they received 1 h worth of study participation credits as compensation.

### Materials and Measures

#### Patient Texts

The texts we used were part of the patient input in a randomized controlled trial that compared a blended internet-based CBT intervention for major depression to an established face-to-face CBT treatment in specialized mental health care settings (Kooistra et al., 2014). Blended, in this case, refers to the integration of an online intervention with weekly face-to-face conversations with an assigned therapist. The online component consisted of 10 modules, typically containing a mixture of psychoeducation and therapeutic exercises. At the end of each module, except for the introductory one, patients were asked for a non-obligatory evaluation on how they experienced the internet module. Therapists were instructed to focus their online communication and feedback on the progress patients made within the intervention, rather than on what had occurred during the face-to-face conversations. The texts were anonymized, and of the texts that occurred more than once, only one was kept in the dataset.

#### Automated Sentiment Analysis

To analyze the texts’ sentiment, we made use of a sentiment mining algorithm that has been tailored toward the Dutch language by using a Dutch grammatical engine and vocabulary (van Breda, 2018). Our choice for this domain-independent algorithm was a pragmatic one, since we are exploring a new domain where tailored algorithms have not yet been developed, and state-of-the-art algorithm’s like those developed by Google (Google, 2018) or IBM (IBM, 2018) have, at the time of writing, not been tailored to the Dutch language. The algorithm has previously been used in studies focusing on crime prediction, where it was used to identify aggressive Twitter messages (Gerritsen and van Breda, 2015), life-style support, where it was used to determine people’s attitude toward a lifestyle goal (van Breda et al., 2012), and a training application for football referees, where it was used to identify the language used by referees during conflicts with football players (Bosse et al., 2017). Though the systems that used the algorithm seemed to have potential following simulation studies and a preliminary evaluation, respectively, they are still under development, and none of the published studies specifically targeted the accuracy of the algorithm. The algorithm accepts strings of text as input, and returns, among others, an overall sentiment value, and scores for 33 specific emotions contained within the text. It uses an advanced form of the bag-of-words approach (Raghavan and Wong, 1986; Zhang et al., 2010) by utilizing lists of words with vectors of weights, and combines this with a rule-based system, operationalized through a grammar detection engine, to analyze the surrounding semantic context of the found sentiment words.

##### Overall sentiment

**FIGURE 1:**
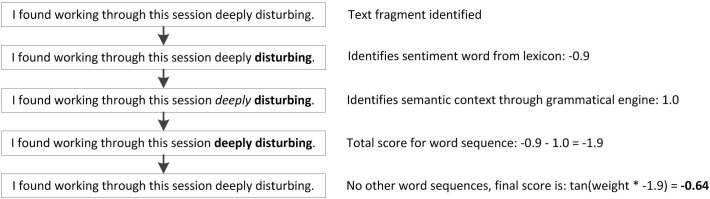
Sentence exemplifying how the algorithm comes to an overall sentiment score for a sentence containing one sentiment word (“disturbing”) from the lexicon combined with a strengthening word (“deeply”) identified by the grammatical engine.

When positive or negative words that are present in the lexicon are identified in the text, the algorithm looks for words that represent the semantic context surrounding them with a grammatical engine. Such words include negation (e.g., “not”), strengthening (e.g., “more”) and weakening (e.g., “less”) words. To all sets of word sequences found a scoring method is applied. The vectors of weights for each word sequence are multiplied with a particular *weight* parameter, and fed to a tangent or logistic activation function. This function normalizes the value to one within the interval [−1, 1], and ensures that extreme scores do not have a disproportionately large effect on the final scores. In the final independent score for positive and negative sentiment, −1 means very negative, and 1 very positive. An example is provided in Figure 1.

##### Emotional expressiveness

For the emotion labels, a similar approach is taken, be it that the text is now searched for words related to that emotion instead of sentiment words in general as a first step. Additionally, it considers emotions either not present or present to a certain extent, and therefore applies a different *weight* parameter to the emotion labels. Scores lay in the interval [0, 1], where 0 means the emotion was not detected, and 1 means the maximum amount of the emotion was detected.

#### Human Sentiment Analysis

**FIGURE 2:**
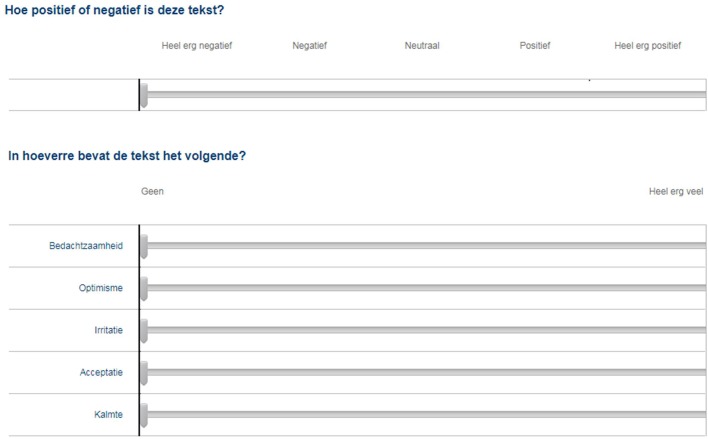
Sliders used by human judges to evaluate texts on sentiment and emotions. Translated from Dutch: (top) *“How positive or negative is this text?”*, answer labels: *very negative; negative; neutral; positive; very positive*, and (bottom) *“To what extent does the text contain the following?”*, answer labels: *none; a whole lot*, five emotions top to bottom: *pensiveness; optimism; annoyance; acceptance; serenity*.

An online questionnaire was designed for the human judges, in which they were presented with a different page for each patient text. For every text, they were asked to evaluate overall sentiment and the presence of five emotions with the use of a slider (see Figure 2).

**FIGURE 3:**
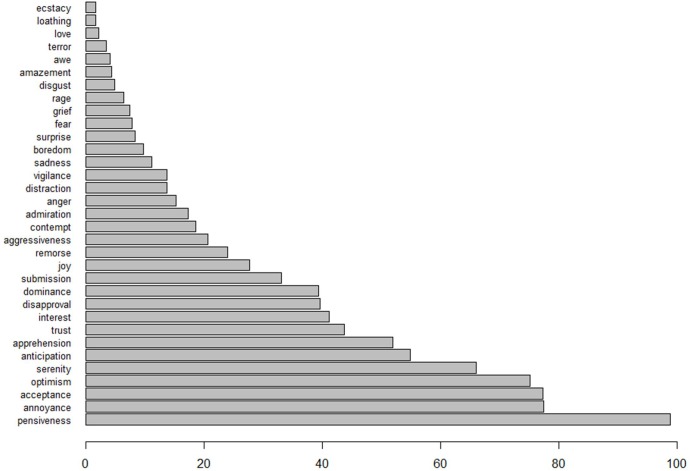
The summed algorithm scores over all 493 texts, with respect to all 33 detectable emotions.

Because asking our participants to evaluate the texts on all 33 emotions considered by the algorithm would have been too burdensome, we chose to focus on the five emotions that the algorithm detected in the texts most often. The summed algorithm scores over all 493 texts are depicted in Figure 3, and the five most prominent emotions we chose to study were *pensiveness*, *annoyance*, *acceptance*, *optimism*, and *serenity*. To prepare the data for the ICC analysis, the ratings provided by human judges were scaled to the same intervals as the automated ratings, i.e. [−1, 1] for overall sentiment, and [0, 1] for the five emotions.

#### Procedure

Participants could register for the study during March and April 2018, and those that did received a personal link to the online questionnaire. At the beginning of the questionnaire they received instructions and signed a digital informed consent, and at the end were given a debriefing with more information about the study.

#### Analysis

To compare algorithm with human judgment, we first calculated the average human rating for every text, which was then used as the value for human judgment. We also converted the numerical values to categorical ones, as practical applications may use a limited set of observable user responses (e.g., positive or negative) to be matched by a limited set of agent responses, for example by responding with positive reinforcement to positive or empathically to negative user input. This is similar to the approach where polarity can be measured either by intensity (continuous) or direction (categorical) (Devitt and Ahmad, 2007). For overall sentiment, three categories were created: *negative* for values smaller than 1, *positive* for values larger than one, and *neutral* for values equal to zero. Because the comparison between humans and algorithm uses the human judges’ average ratings, which are unlikely to be equal to zero, we defined *neutral* sentiment as any value equal to or larger than −0.1, and equal to or smaller than 0.1. For the different emotions, two categories were created: *not present* for values equal to or smaller than 0.1, and *present* for values larger than 0.1.

We used the R statistical software (R Core Team, 2016), extended with the *psych* (Revelle, 2018) and *irr* (Gamer et al., 2012) packages to calculate intra-class correlations (ICC), Cohen’s kappa (κ), and Krippendorff’s alpha (α).

##### Intra-class correlation

ICCs can be used to assess IRR on items with a continuous scale, and are also applicable to cases with more than two raters or missing values. Values range between 0 and 1, with values less than 0.5 indicative of poor, values between 0.5 and 0.75 of moderate, values between 0.75 and 0.9 of good, and values greater than 0.9 of excellent reliability (Shrout and Fleiss, 1979). In our analysis under the continuous interpretation of the results, we used ICC(1,1) to calculate agreement among humans, accounting for one-way random effects caused by our research setup (different judges from one population rate each item), and ICC(3,1) to calculate agreement between the algorithm and human judges, accounting for two-way mixed effects (Koo and Li, 2016).

##### Cohen’s kappa

Cohen’s kappa can be used to assess IRR on items with a categorical scale (Cohen, 1960), and a weighted κ can be calculated to account for ordered categories. Values range between −1 and 1, with values lower than 0 indicative of no, values between 0 and 0.4 of slight to fair, values between 0.41 and 0.6 of moderate, values between 0.61 and 0.8 of substantial, and values between 0.81 and 1 of almost perfect reliability (Landis and Koch, 1977). In our analysis we used the weighted κ to calculate agreement between the algorithm and humans under the categorical interpretation of results.

##### Krippendorff’s alpha

Krippendorff’s alpha can also be used to assess IRR on items with a categorical scale, but contrary to Cohen’s Kappa is able to deal with missing values (Hayes and Krippendorff, 2007). Values range between −1 and 1, and can be considered reliable if larger than 0.8, with values larger than 0.67 allowing tentative conclusions to be drawn (Artstein and Poesio, 2008). We used α to calculate agreement among human judges under the categorical interpretation of results, since there were many missing values due to every item being rated by a limited set of judges.

## Results

### Descriptives

A total of 52 participants completed the experiment. Every text received on average *M* = 8.1 (range 4–10) human evaluations, and a total of 3900 evaluations was provided. Table 1 contains the summary statistics of the averaged human and algorithm evaluations with respect to sentiment and the five emotions. It shows a smaller range of values for human evaluation, caused by taking the average values, and consequently a smaller standard deviation as well. For human evaluation, median values did not differ much from mean values, which means there was no reason to use the median in our analysis.

**Table 1:** Summary statistics of averaged human (*M* = 8.1 evaluations per text) and algorithm judgment of the *N* = 493 total number of texts.

	*M*	Min.	Max.	*SD*
**Sentiment**				
Human judges	0.00	−0.71	0.67	0.30
Algorithm	0.04	−0.97	0.99	0.44
**Pensiveness**				
Human judges	0.47	0.01	0.78	0.15
Algorithm	0.47	0.00	1.00	0.44
**Annoyance**				
Human judges	0.40	0.04	0.81	0.17
Algorithm	0.39	0.00	1.00	0.44
**Optimism**				
Human judges	0.37	0.01	0.86	0.20
Algorithm	0.40	0.00	1.00	0.42
**Acceptance**				
Human judges	0.25	0.02	0.86	0.17
Algorithm	0.40	0.00	1.00	0.42
**Serenity**				
Human judges	0.38	0.02	0.79	0.16
Algorithm	0.37	0.00	1.00	0.41

**FIGURE 4:**
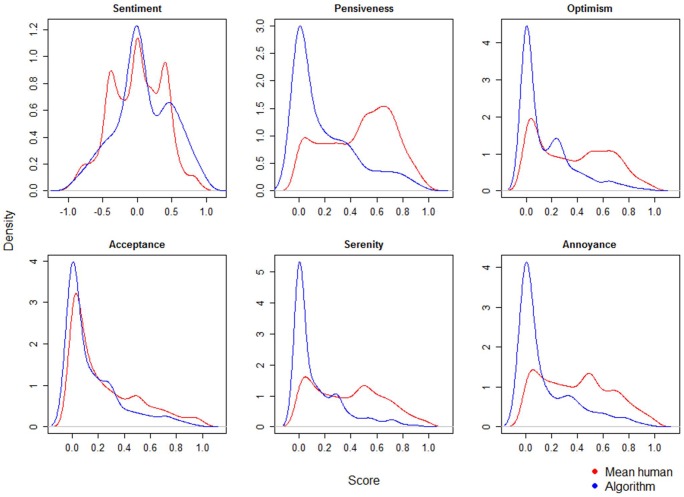
Probability distributions of human and algorithm evaluations.

Figure 4 depicts the distributions of the ratings, with those for sentiment approaching a normal distribution, and those for the different emotions showing right-skewed distributions with one large peak (no emotion detected) for the algorithm, and varying distributions for human evaluation.

**FIGURE 5:**
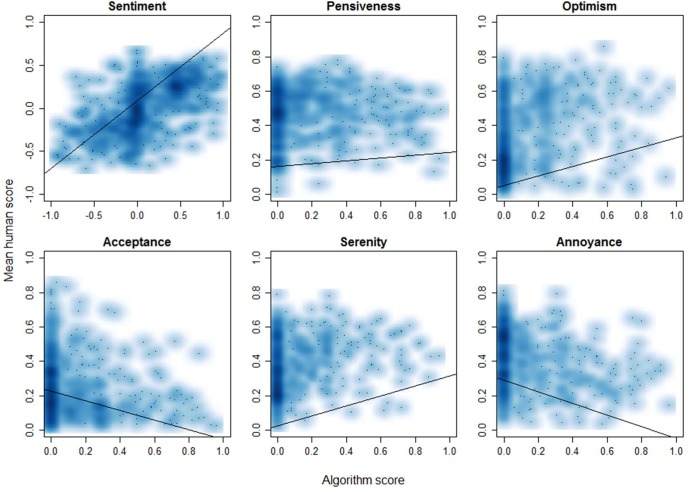
Scatterplots of the algorithm versus the mean human scores for all of the 493 patient texts, including a line representing the linear fit model.

Figure 5 shows scatter plots of the raw data including a line representing a linear fit model. On visual inspection, human and algorithm evaluation have the best correlation for sentiment, while for acceptance and annoyance they are negatively correlated.

### Agreement Among Human Raters

#### Continuous Interpretation

As shown in Table 2, an ICC(1,1) analysis revealed a moderate IRR among human raters with regard to sentiment (ICC = 0.58, CI = 0.54–0.61, *F*(492,25143) = 71, *p* < 0.01). IRR among human raters with regard to the different emotions was overall poor, and only with regard to optimism did the judges get close to moderate agreement.

**Table 2:** Results of intra-class correlation on human-human agreement.

	Intraclass correlation	95% confidence interval		*F*-test with true value 0			
		Lower bound	Upper bound	Value	df1	df2	significant
Sentiment	0.58	0.54	0.61	71	492	25143	0.00
Pensiveness	0.22	0.20	0.25	16	492	25143	0.00
Annoyance	0.28	0.16	0.31	21	492	25143	0.00
Optimism	0.46	0.43	0.50	46	492	25143	0.00
Acceptance	0.34	0.32	0.38	28	492	25143	0.00
Serenity	0.24	0.21	0.26	17	492	25143	0.00

#### Categorical Interpretation

For the categorical human-human interrater agreement, a Krippendorff’s alpha of α = 0.51 was found, which indicates moderate agreement similar to ICC results for the continuous interpretation of ratings. Agreement with respect to the emotions was poor. An overview is provided in Table 3.

**Table 3:** Krippendorff’s alpha values for human-human agreement.

Emotion	α
Sentiment	0.51
Pensiveness	0.17
Annoyance	0.20
Optimism	0.28
Acceptance	0.23
Serenity	0.17

### Agreement Between Human Judges and Algorithm

#### Continuous Interpretation

An ICC(3,1) analysis revealed a moderate IRR between human raters and the algorithm with regard to sentiment (ICC = 0.55, CI = 0.48–0.61, *F*(492,492) = 3.4, *p* < 0.01). IRR between the human raters and the algorithm with regard to the different emotions was poor (see Table 4).

**Table 4:** ICC values for human-algorithm agreement.

	Intraclass correlation	95% confidence interval		*F*-test with true value 0			
		Lower bound	Upper bound	Value	df1	df2	significant
Sentiment	0.55	0.48	0.61	3.4	492	492	0.00
Pensiveness	0.12	0.03	0.21	1.3	492	492	0.00
Annoyance	0.00	−0.09	0.09	1	492	492	0.5
Optimism	0.23	0.14	0.31	1.6	492	492	0.00
Acceptance	0.00	−0.09	0.09	1	492	492	0.5
Serenity	0.14	0.06	0.23	1.3	492	492	0.00

#### Categorical Interpretation

A weighted Cohen’s kappa was calculated, indicating there was moderate agreement with regard to sentiment, κ = 0.58 (95% CI, 0.52 to 0.63), *p* = 0.05. As is illustrated in Table 5, similar to the agreement observed when considering continuous data, the correlation between human and algorithm evaluation remained poor.

**Table 5:** Cohen’s kappa values for human-algorithm agreement.

Emotion	κ	95% CI
Sentiment	0.58	0.52 to 0.63
Pensiveness	0.01	−0.01 to 0.03
Annoyance	−0.01	−0.04 to 0.02
Optimism	0.09	0.03 to 0.14
Acceptance	−0.07	−0.14 to 0.00
Serenity	0.03	0.00 to 0.06

Tables 6, 7 give an overview of the accuracy of the algorithm compared to human judgment for both overall sentiment (65.92%) and the emotion with the highest κ-value, optimism (54.8%).

**Table 6:** Comparison of the average human judges’ and algorithm’s evaluations with respect to sentiment.

		Algorithm		
		Negative	Neutral	Positive
Human Average	Negative	144	15	38
	Neutral	30	19	41
	Positive	25	19	162

**Table 7:** Comparison of the average human judges’ and algorithm’s evaluations with respect to optimism.

		Algorithm	
		Not present	Present
Human Average	Not present	32	12
	Present	211	238

## Discussion

### Principal Results

The aim of this study was to investigate how well humans and a sentiment analysis algorithm agree on evaluating the overall sentiment and presence of five emotions in patient input in an iCBT intervention for depression. Regarding sentiment, human-human agreement was moderate, both under the continuous (ICC = 0.58) and categorical (α = 0.51) interpretations. Algorithm-human agreement was moderate as well, again both under the continuous (ICC = 0.55) and categorical (κ = 0.58) interpretations. With respect to the different emotions, human-human agreement was overall poor. Most consensus was achieved on optimism (ICC = 0.46), which could be considered ‘fair’ agreement under an alternative interpretation (Cicchetti, 1994). Human-algorithm agreement on the presence of emotions was poor as well, with the highest agreement once again being achieved for optimism (ICC = 0.23), and agreement on the other emotions being negligible. In two cases, for acceptance and annoyance, the correlations even appeared to be slightly negative, as can be observed in Figure 5. The interpretation of low human-algorithm agreement on the presence of emotions deserves some caution though, as the low human-human agreement for the presence of emotions compared to the moderate human-human agreement for overall sentiment makes for a less solid benchmark.

When we look at performance of the algorithm compared to human judgment under the categorical interpretation in terms of accuracy (65.92%) it seemed reasonable, but higher values have been reported. Some examples of higher accuracies are 70.2% for a corpus of software reviews when comparing an algorithm to three judges (Aggarwal and Singh, 2013), and around 88% for a corpus of 1000 comments on Youtube videos related to anorexia (Oksanen et al., 2015), and some with lower accuracies are 47% for a corpus of news reports on a potential hostile take-over of an airline (Devitt and Ahmad, 2007), and 65.7% for 447 subjective statements in a corpus of 10 news articles (Wilson et al., 2005). Algorithms for the Dutch language have been benchmarked sparsely, an example being 70.0% for a corpus of 60 social media texts (Tromp, 2011). Since there were an average of *M* = 8.1 human ratings per text, and the mean human rating for each text was used as a benchmark, calculating an accuracy score for human-human agreement is not straightforward. Nevertheless, we can say that there was relatively low agreement among human judges, as Wilson et al. in their study using 10 news articles, for example, reported an accuracy of 82%, and IRR of κ = 0.72 with regard to evaluating sentiment (Wilson et al., 2005).

With respect to overall sentiment, this means that the algorithm does about as well at discerning between positive and negative sentiment as would a random human judge from our population of judges, agreement being moderate in both conditions. The same thing held for the evaluation of emotions, as agreement was poor in both conditions. This could mean that it is equally difficult for humans to evaluate the texts in our domain, or that the emotions we chose are hard to apply to our texts, but it could also indicate that the texts are too ambiguous. Even though they were supposed to be about patients’ opinions on the treatment module they had just finished, they were not necessarily limited to this topic. Since we used texts from a blended-care intervention, i.e., patients spoke to their therapists face-to-face as well, it is possible that patients took the review as an opportunity to inform their therapists of other things as well, such as significant life events that took place. On the other hand, however, it is also possible that it was the other way around, with patients preserving emotionally laden topics for the face-to-face meetings. Both factors may have been at play, as texts varied a lot in terms of length and content, with input ranging from the Dutch abbreviation for ‘not applicable’ and concise evaluations of the module (e.g., “This session gave me a good insight into different activities”), to extensive reports on their current mental state and recent life events, such as the impact of family events on their mood. Despite the ambiguous nature of some texts, we chose to use them as they were, because manual filtering of the texts would not happen in the practical application we envision either.

Furthermore, we chose to benchmark the algorithm versus the average scores of human judges as a “gold standard.” These human judges, however, were first year psychology students, and although these can be considered to have at least some affinity with our domain, actual therapists or people with experience in providing guidance to iCBT as human judges may have provided data more closely resembling the evaluation of judgment in clinical practice. However, that this would not necessarily mean higher inter-rater agreement, is exemplified by two studies into inter-rater agreement among clinicians when identifying overt problems and underlying schemas of CBT patients (Persons et al., 1995; Persons Jacqueline and Bertagnolli, 1999). Even though the judges in these studies were asked to rate patient interviews instead of short text fragments, the studies showed that clinicians can have considerable trouble agreeing on patient data as well. This was especially true when individual judges were concerned instead of a group average, and little evidence was found that a higher level of expertise improved judgment.

### Future Research

With average human judgment as our “gold standard,” and the algorithm performing about as well as a random human judge from our population, the algorithm could be applied for overall sentiment analysis of patient input in a practical application if we consider this “gold standard” as good enough. This is not the case for the specific emotions, since both human-human and human-algorithm agreement were low at best. As briefly described in the introduction, our aim is to develop an ECA that can support people who are working through an iCBT intervention for depression. Because of the technological limitations in interpreting and producing natural language automatically, we envision a tree-based dialog approach in which users choose their responses from a menu, as the safest way to structure a conversation. The limited number of pathways that represent all different possible conversations are relatively easy to understand and finite, which is important if we want mental health specialists to review the dialogs. Moreover, tree-based dialog approaches allow for the use of threshold values of parameters (e.g., negative or positive sentiment) to determine which path to take through a conversation, and therefore lend themselves well to our domain.

Considering such a supportive ECA, applying the algorithm to descriptions of how patients experienced intervention modules, could allow it to determine whether this was positive or negative, and to consequently provide automated personalized feedback through positive reinforcement (e.g., “It seemed to me that you liked working through the previous module, good luck with the next one!”) or a more empathic response (e.g., “I noticed you finished the previous module, even though you did not seem to like it much. Impressive, keep up the good work!”) respectively. Given the relatively large margin of error (around 1 in 3 evaluations would be wrong given our 65.92% accuracy), however, it seems imperative to build in a security mechanism to avoid incoherent communication by the ECA. A possibility is to first ask a user for confirmation (e.g., “It seemed to me that you did not like the previous module. Is that correct?”), and only then continue with the appropriate motivational message. Incorporating such a mechanism in a dialog may seem superfluous, as the ECA could also ask a patient directly about his or her opinion on the previous module, however, an important aspect of providing support is for a guiding person to show that he or she has actively looked at what a patient has been doing, for example through summarizing or reflecting (Mol et al., 2018), rather than asking things that a patient has already told the guiding person indirectly through the intervention.

We could also look to apply the algorithm to, or validate it against other user input, such as responses to questions about willingness and readiness to change, or to questions about how their homework exercises (e.g., experimenting with certain behaviors) are going. By targeting the right set of patient input, it may even be possible to determine a general mood of the patient by comparing the use of language over time.

A different angle of future work would be to tune the algorithm to our iCBT domain. Now that we have a large dataset of human evaluations, a bottom-up approach with machine learning could be used to tailor the algorithm toward the domain-specific vocabulary. Although to our knowledge, this has not been done in the context of sentiment analysis in iCBT, machine learning has been successfully applied in the domain of mental health disorders. For example, data-driven approaches outperformed human judgment in the prediction of iCBT treatment success (Amethier and Haggren, 2018), and non-fatal suicide attempts (Walsh et al., 2018). A comparison with these approaches deserves some caution, however, firstly because the data which was used to train the algorithms consisted of demographic and psychometric information rather than text. Secondly, treatment success, as well as non-fatal suicide attempts, are more solid benchmarks than human judgment in this study. If we aim to outperform a gold standard, in our case one of judging sentiment contained in a text, a more objective benchmark may be required. In light of the sparse evidence regarding the validity of human judgment as a benchmark, it could be interesting to compare an algorithm’s performance to patients’ own judgment.

## Conclusion

Sentiment analysis could be a promising tool with which to enhance the personalization of automated feedback in iCBT interventions, for example through conversations with a supportive ECA. Our study showed that an existing algorithm for the Dutch language performed about equally well as a randomly chosen human judge at distinguishing between negative, neutral, and positive sentiment present in free-text patient input. The algorithm performed poorly at evaluating the presence of specific emotions, but the human judges, even though they were more consistent with each other than with the algorithm, performed poorly as well in terms of inter-rater agreement. This means that it may be worthwhile to validate the algorithm against a potentially more solid benchmark, such as patients’ own judgment. If we were to consider the level of human-human agreement reported in this study to be the gold standard for our domain, automated sentiment analysis could be considered applicable. However, given the somewhat higher accuracy scores found in the analysis of, for example, social media messages or product reviews, it may be worthwhile to build in a security mechanism that confirms the automated analysis if it were used in practice, or to tailor an algorithm to the domain of iCBT interventions.

## Author Contributions

SP: substantial contribution to every aspect of the manuscript, critical revision, and final approval and agrees to be accountable. JR: substantial contribution to design and conception, analysis, critical revision, and final approval and agrees to be accountable. WvB: substantial contribution to the acquisition of data, critical revision, and final approval and agrees to be accountable. HR: substantial contribution to design and conception, critical revision, and final approval and agrees to be accountable. TB: substantial contribution to design and conception, critical revision, and final approval and agrees to be accountable.

## Conflict of Interest Statement

The authors declare that the research was conducted in the absence of any commercial or financial relationships that could be construed as a potential conflict of interest.
